# Supplementary material for: First Examples of s-Metal Complexes with Subporphyrazine and Its Phenylene-Annulated Derivatives: DFT Calculations
Source: Int J Mol Sci. 2024 Jun 24;25(13):6897. doi: 10.3390/ijms25136897 (PMC11241550; doi:10.3390/ijms25136897)
Supplement: Supplementary file 1 [file ijms-25-06897-s001.zip › Supplementary Materials.pdf]

Supplementary Materials

First Examples of s-Metal Complexes with Subporphyrazine and Its Phenylene-Annulated Derivatives: DFT Calculations

Denis V. Chachkov, Oleg V. Mikhailov and Georgiy V. Girichev

Content Page

1. Figure S1. Molecular structures of the beryllium complexes with subporphyrazine and its mono-, di- and tri[benzo]-annelated derivatives obtained by using quantum-chemical calculation by DFT M062X/def2TZVP method: *a*: [BeSP], *b*: [BeMBSP], *c*: [BeDBSP], *d*: [BeTBSP] 2

2. Figure S2. The pictures of HOMO and LUMO in the [Be**SP**], [Be**MBSP**], [Be**DBSP**] and [Be**TBSP**] complexes (ground state – spin singlet, *Ms* = 1) according to the DFT M062X/def2TZVP method. The energies values of the given MOs (in brackets) are expressed in eV. 3

3. NBO Analysis Data (Complex[BeSP])…………………… ……………… 4

- B3PW91/TZVP…………………………………………………….. 4
- M062X/def2TZVP………………………………………………… 6
- B3PW91/TZVP…………………………………………….......... 8
- M062X/Def2TZVP………………………………………………… 10
- B3PW91/TZVP…………………………………………………….. 12
- M062X/Def2TZVP………………………………………………… 14
- B3PW91/TZVP…………………………………………………….. 16
- M062X/Def2TZVP………………………………………………… 18

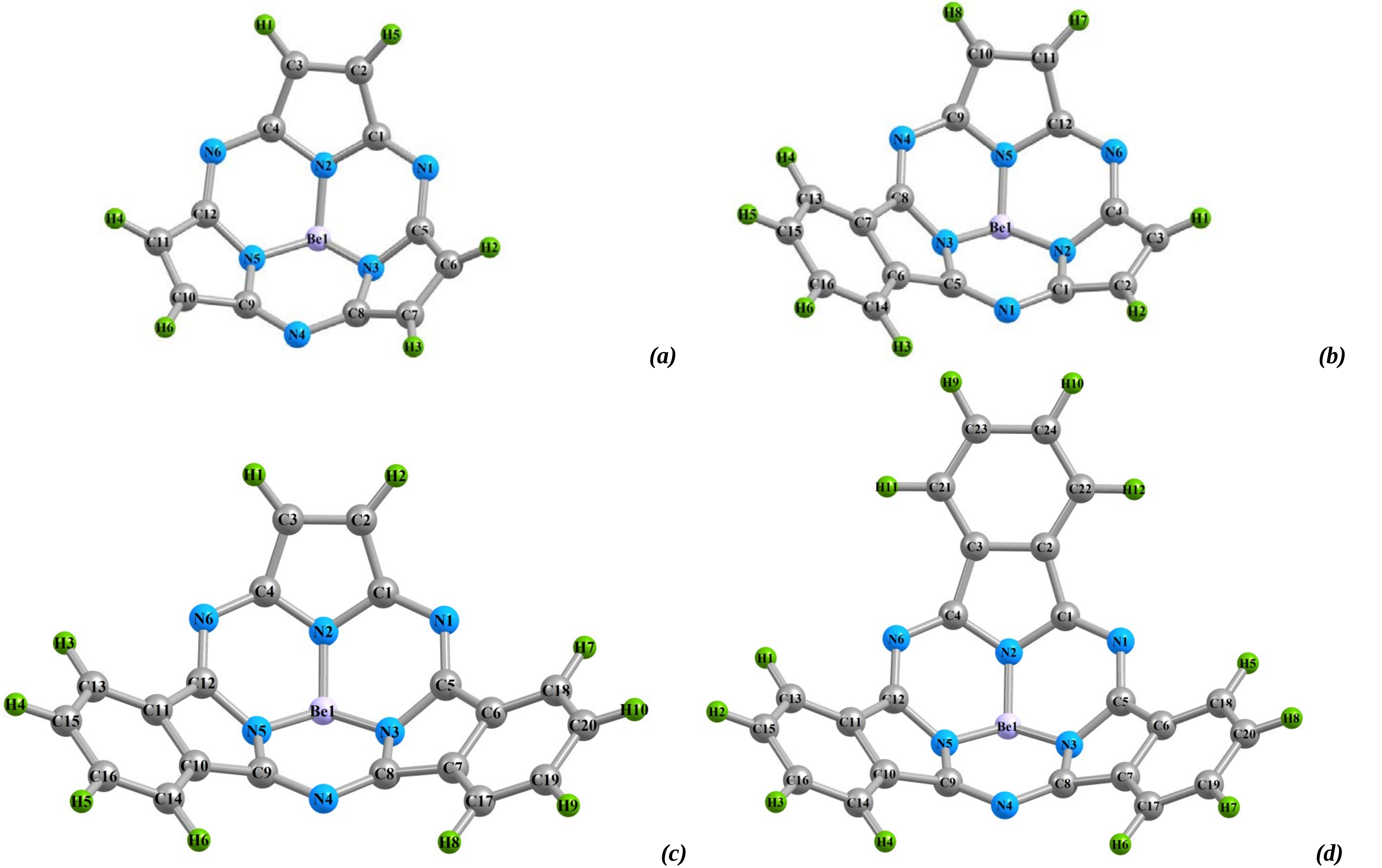

Figure S1. Molecular structures of the beryllium complexes with subporphyrazine and its mono-, di- and tri[benzo]-annelated derivatives obtained by using quantum-chemical calculation by DFT M062X/def2TZVP method: *a*: [Be**SP**], *b*: [Be**MBSP**], *c*: [Be**DBSP**], *d*: [Be**TBSP**].

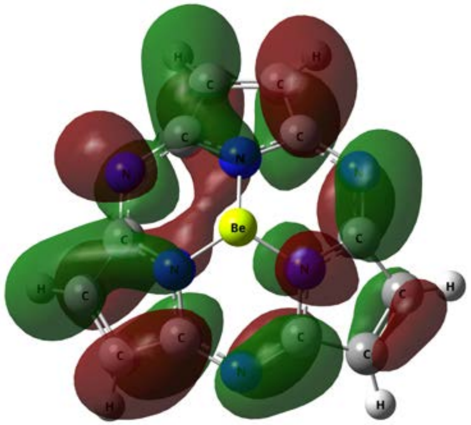	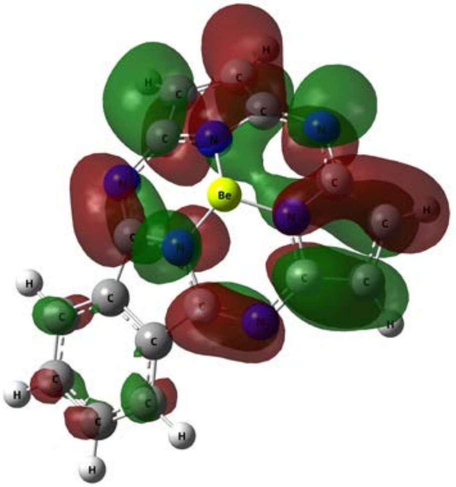	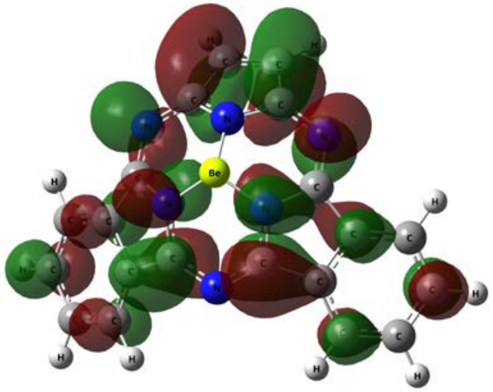	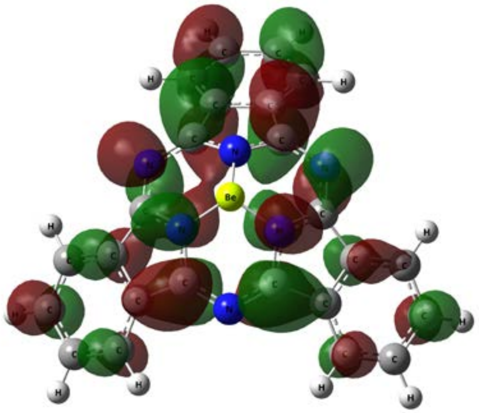
LUMO (**-1.973**)	LUMO (**-2.050**)	LUMO (**-1.986**)	LUMO (**-1.814**)
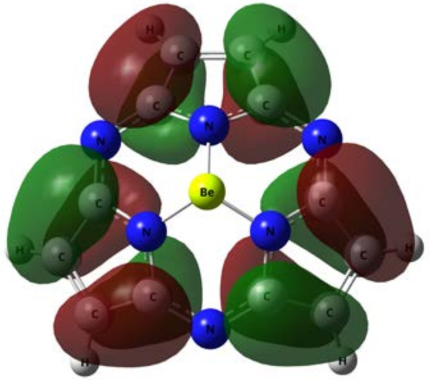	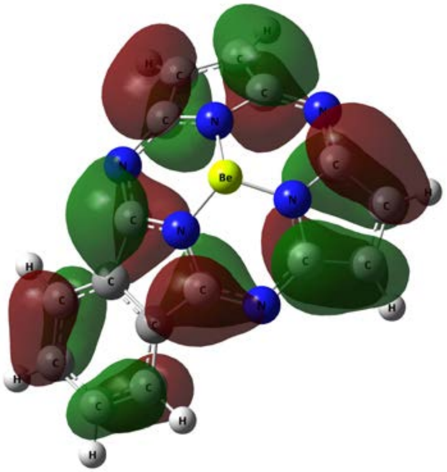	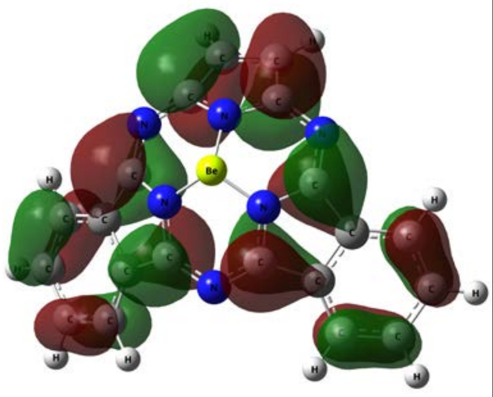	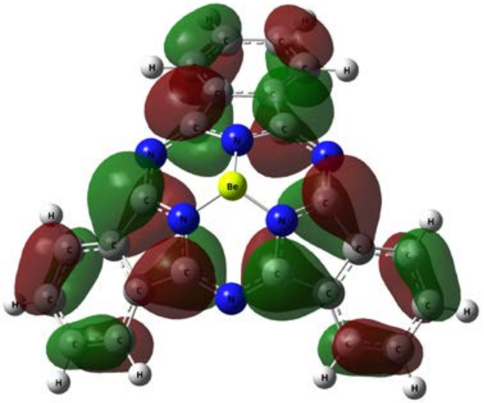
HOMO (**-6.832**)	HOMO (**-6.592**)	HOMO (**-6.351**)	HOMO (**-6.037**)
[Be**SP**]	[Be**MBSP**]	[Be**DBSP**]	[Be**TBSP**]

Figure S2. The pictures of HOMO and LUMO in the [Be**SP**], [Be**MBSP**], [Be**DBSP**] and [Be**TBSP**] complexes (ground state – spin singlet, M_S = 1) according to the DFT M062X/def2TZVP method. The energies values of the given MOs (in brackets) are expressed in eV.

NBO Analysis Data (Complex [BeSP])

B3PW91/TZVP

ΔE(multipl.=1) = 0.0 кДж/моль
ΔE(multipl.=3) = 159.0 кДж/моль
Alpha occupied eigenvalues (highest) = -6.1010262 eV
Alpha virtual eigenvalues (lowest) = -2.8390914 eV

<S**2>= 0.0000

Summary of Natural Population Analysis:

Atom	No	Natural Charge	Natural Population Core	Valence	Rydberg	Total
N	1	-0.40051	1.99932	5.38573	0.01545	7.40051
C	2	0.33186	1.99922	3.64473	0.02418	5.66814
C	3	-0.22714	1.99910	4.21491	0.01313	6.22714
N	4	-0.82790	1.99937	5.80616	0.02237	7.82790
C	5	-0.22715	1.99910	4.21492	0.01313	6.22715
C	6	0.33183	1.99922	3.64476	0.02418	5.66817
H	7	0.23584	0.00000	0.76284	0.00132	0.76416
C	8	0.33186	1.99922	3.64473	0.02418	5.66814
C	9	-0.22714	1.99910	4.21491	0.01313	6.22714
N	10	-0.82790	1.99937	5.80616	0.02237	7.82790
C	11	-0.22714	1.99910	4.21492	0.01313	6.22714
H	12	0.23584	0.00000	0.76284	0.00132	0.76416
C	13	0.33183	1.99922	3.64476	0.02418	5.66817
H	14	0.23584	0.00000	0.76284	0.00132	0.76416
N	15	-0.40052	1.99932	5.38574	0.01545	7.40052
C	16	0.33186	1.99922	3.64473	0.02419	5.66814
C	17	-0.22714	1.99910	4.21492	0.01313	6.22714
N	18	-0.82788	1.99937	5.80613	0.02238	7.82788

C	19	-0.22714	1.99910	4.21492	0.01313	6.22714
C	20	0.33186	1.99922	3.64473	0.02419	5.66814
H	21	0.23584	0.00000	0.76284	0.00132	0.76416
H	22	0.23584	0.00000	0.76284	0.00132	0.76416
H	23	0.23584	0.00000	0.76284	0.00132	0.76416
N	24	-0.40052	1.99932	5.38574	0.01545	7.40052
Be	25	1.64193	1.99829	0.27451	0.08526	2.35807
* Total *		0.00000	37.98429	85.58516	0.43055	124.00000

NBO Analysis Data (Complex [BeSP])

M062X/def2TZVP

ΔE(multipl.=1) = 0.0 кДж/моль
ΔE(multipl.=3) = 157.3 кДж/моль
Alpha occupied eigenvalues (highest) = -6.832431 eV
Alpha virtual eigenvalues (lowest) = -1.9729971 eV

<S**2>= 0.0000

Summary of Natural Population Analysis:

Atom	No	Natural Charge	Natural Population Core	Valence	Rydberg	Total
N	1	-0.41569	1.99933	5.38657	0.02978	7.41569
C	2	0.34411	1.99924	3.63140	0.02525	5.65589
C	3	-0.22676	1.99910	4.20912	0.01854	6.22676
N	4	-0.84936	1.99935	5.81529	0.03471	7.84936
C	5	-0.22676	1.99910	4.20912	0.01854	6.22676
C	6	0.34412	1.99924	3.63139	0.02525	5.65588
H	7	0.23553	0.00000	0.76340	0.00106	0.76447
C	8	0.34412	1.99924	3.63138	0.02525	5.65588
C	9	-0.22673	1.99910	4.20908	0.01854	6.22673
N	10	-0.84934	1.99935	5.81528	0.03471	7.84934
C	11	-0.22677	1.99910	4.20912	0.01854	6.22677
H	12	0.23553	0.00000	0.76341	0.00106	0.76447
C	13	0.34412	1.99924	3.63138	0.02525	5.65588
H	14	0.23555	0.00000	0.76339	0.00106	0.76445
N	15	-0.41567	1.99933	5.38655	0.02979	7.41567
C	16	0.34408	1.99924	3.63143	0.02525	5.65592
C	17	-0.22676	1.99910	4.20911	0.01854	6.22676
N	18	-0.84933	1.99935	5.81527	0.03471	7.84933

C	19	-0.22677	1.99910	4.20912	0.01855	6.22677
C	20	0.34413	1.99924	3.63138	0.02525	5.65587
H	21	0.23553	0.00000	0.76341	0.00106	0.76447
H	22	0.23554	0.00000	0.76339	0.00106	0.76446
H	23	0.23554	0.00000	0.76340	0.00106	0.76446
N	24	-0.41569	1.99933	5.38658	0.02978	7.41569
Be	25	1.67771	1.99831	0.25159	0.07239	2.32229
* Total *		0.00000	37.98443	85.48055	0.53502	124.00000

NBO Analysis Data (Complex [BeMBSP])

B3PW91/TZVP

ΔE(multipl.=1) = 0.0 кДж/моль
ΔE(multipl.=3) = 140.6 кДж/моль
Alpha occupied eigenvalues (highest) = -5.8360008 eV
Alpha virtual eigenvalues (lowest) = -2.8627641 eV

<S2>= 0.0000**

Summary of Natural Population Analysis:

Atom	No	Natural Charge	Natural Population: Core	Valence	Rydberg	Total
N	1	-0.41868	1.99932	5.40364	0.01571	7.41868
C	2	0.33633	1.99921	3.64022	0.02424	5.66367
C	3	-0.24131	1.99910	4.22894	0.01327	6.24131
N	4	-0.82837	1.99937	5.80597	0.02302	7.82837
C	5	-0.22278	1.99911	4.21023	0.01344	6.22278
C	6	0.31369	1.99921	3.66265	0.02445	5.68631
H	7	0.23456	0.00000	0.76396	0.00147	0.76544
C	8	0.37509	1.99918	3.60137	0.02435	5.62491
C	9	-0.07896	1.99904	4.06272	0.01721	6.07896
N	10	-0.81001	1.99940	5.78594	0.02468	7.81001
C	11	-0.07893	1.99904	4.06268	0.01720	6.07893
C	12	0.37501	1.99918	3.60145	0.02435	5.62499
N	13	-0.41859	1.99932	5.40355	0.01571	7.41859
C	14	0.33616	1.99921	3.64039	0.02424	5.66384
C	15	-0.24130	1.99910	4.22892	0.01327	6.24130
N	16	-0.82830	1.99937	5.80590	0.02303	7.82830
C	17	-0.22284	1.99911	4.21030	0.01344	6.22284
C	18	0.31364	1.99921	3.66271	0.02445	5.68636
H	19	0.23474	0.00000	0.76400	0.00126	0.76526

N	20	-0.38852	1.99933	5.37286	0.01633	7.38852
Be	21	1.64368	1.99828	0.27407	0.08397	2.35632
C	22	-0.17479	1.99912	4.16181	0.01387	6.17479
C	23	-0.17478	1.99912	4.16180	0.01387	6.17478
C	24	-0.20118	1.99924	4.18786	0.01408	6.20118
C	25	-0.20120	1.99924	4.18787	0.01408	6.20120
H	26	0.23124	0.00000	0.76717	0.00159	0.76876
H	27	0.23124	0.00000	0.76717	0.00159	0.76876
H	28	0.21793	0.00000	0.78095	0.00112	0.78207
H	29	0.21793	0.00000	0.78095	0.00112	0.78207
H	30	0.23455	0.00000	0.76398	0.00147	0.76545
H	31	0.23473	0.00000	0.76401	0.00126	0.76527
* Total *		-0.00000	45.98080	103.51605	0.50316	150.00000

NBO Analysis Data (Complex [BeMBSP])

M062X/Def2TZVP

ΔE(multipl.=1) = 0.0 кДж/моль
ΔE(multipl.=3) = 138.9 кДж/моль
Alpha occupied eigenvalues (highest) = -6.5916225 eV
Alpha virtual eigenvalues (lowest) = -2.0502735 eV

<S**2>= 0.0000

Summary of Natural Population Analysis:

Atom	No	Natural Charge	Natural Population Core	Valence	Rydberg	Total
N	1	-0.43831	1.99932	5.40905	0.02994	7.43831
C	2	0.35173	1.99922	3.62406	0.02499	5.64827
C	3	-0.24691	1.99911	4.22945	0.01836	6.24691
N	4	-0.84725	1.99935	5.81295	0.03494	7.84725
C	5	-0.21750	1.99911	4.20008	0.01831	6.21750
C	6	0.31286	1.99922	3.66298	0.02493	5.68714
H	7	0.23411	0.00000	0.76483	0.00105	0.76589
C	8	0.39807	1.99921	3.57872	0.02399	5.60193
C	9	-0.08215	1.99903	4.06278	0.02033	6.08215
N	10	-0.83754	1.99938	5.80335	0.03480	7.83754
C	11	-0.08221	1.99903	4.06284	0.02033	6.08221
C	12	0.39818	1.99921	3.57861	0.02400	5.60182
N	13	-0.43838	1.99933	5.40913	0.02993	7.43838
C	14	0.35200	1.99922	3.62380	0.02498	5.64800
C	15	-0.24691	1.99911	4.22944	0.01836	6.24691
N	16	-0.84727	1.99935	5.81297	0.03495	7.84727
C	17	-0.21733	1.99911	4.19991	0.01831	6.21733
C	18	0.31301	1.99922	3.66284	0.02493	5.68699

H	19	0.23465	0.00000	0.76435	0.00100	0.76535
N	20	-0.39211	1.99933	5.36274	0.03005	7.39211
Be	21	1.67885	1.99829	0.25108	0.07179	2.32115
C	22	-0.17360	1.99913	4.15677	0.01770	6.17360
C	23	-0.17362	1.99913	4.15679	0.01770	6.17362
C	24	-0.19923	1.99926	4.18210	0.01788	6.19923
C	25	-0.19920	1.99926	4.18207	0.01788	6.19920
H	26	0.23121	0.00000	0.76760	0.00119	0.76879
H	27	0.23122	0.00000	0.76760	0.00119	0.76878
H	28	0.21743	0.00000	0.78164	0.00094	0.78257
H	29	0.21743	0.00000	0.78164	0.00094	0.78257
H	30	0.23412	0.00000	0.76483	0.00105	0.76588
H	31	0.23467	0.00000	0.76433	0.00100	0.76533
* Total *		0.00000	45.98094	103.41131	0.60775	150.00000

<u>NBO Analysis Data (Complex [BeDBSP])</u>

B3PW91/TZVP

ΔE(multipl.=1) = 0.0 кДж/моль
ΔE(multipl.=3) = 131.1 кДж/моль
Alpha occupied eigenvalues (highest) = -5.5853967 eV
Alpha virtual eigenvalues (lowest) = -2.7688896 eV

<S2>= 0.0000**

Summary of Natural Population Analysis:

Atom	No	Natural Charge	Natural Population Core	Valence	Rydberg	Total
N	1	-0.40452	1.99932	5.38929	0.01590	7.40452
C	2	0.31302	1.99918	3.66326	0.02454	5.68698
C	3	-0.23894	1.99911	4.22624	0.01359	6.23894
N	4	-0.82996	1.99938	5.80707	0.02351	7.82996
C	5	-0.23894	1.99911	4.22624	0.01359	6.23894
C	6	0.31302	1.99918	3.66326	0.02454	5.68698
H	7	0.23303	0.00000	0.76552	0.00145	0.76697
C	8	0.35535	1.99918	3.62140	0.02407	5.64465
C	9	-0.07355	1.99904	4.05748	0.01702	6.07355
N	10	-0.80784	1.99940	5.78405	0.02439	7.80784
C	11	-0.08622	1.99904	4.07010	0.01708	6.08622
C	12	0.38163	1.99917	3.59510	0.02410	5.61837
N	13	-0.43648	1.99933	5.42130	0.01585	7.43648
C	14	0.38163	1.99917	3.59510	0.02410	5.61837
C	15	-0.08622	1.99904	4.07010	0.01708	6.08622
N	16	-0.80784	1.99940	5.78405	0.02439	7.80784
C	17	-0.07355	1.99904	4.05748	0.01702	6.07355
C	18	0.35535	1.99918	3.62140	0.02407	5.64465

H	19	0.23303	0.00000	0.76552	0.00145	0.76697
N	20	-0.40452	1.99932	5.38929	0.01590	7.40452
Be	21	1.64514	1.99827	0.27351	0.08308	2.35486
C	22	-0.17720	1.99912	4.16420	0.01388	6.17720
C	23	-0.17355	1.99912	4.16061	0.01383	6.17355
C	24	-0.19998	1.99924	4.18664	0.01410	6.19998
C	25	-0.20570	1.99924	4.19229	0.01417	6.20570
H	26	0.23074	0.00000	0.76767	0.00159	0.76926
H	27	0.21727	0.00000	0.78161	0.00112	0.78273
H	28	0.21732	0.00000	0.78155	0.00113	0.78268
H	29	0.22979	0.00000	0.76860	0.00161	0.77021
C	30	-0.17355	1.99912	4.16061	0.01383	6.17355
C	31	-0.17720	1.99912	4.16420	0.01388	6.17720
C	32	-0.20570	1.99924	4.19229	0.01417	6.20570
C	33	-0.19998	1.99924	4.18664	0.01410	6.19998
H	34	0.23074	0.00000	0.76767	0.00159	0.76926
H	35	0.22979	0.00000	0.76860	0.00161	0.77021
H	36	0.21732	0.00000	0.78155	0.00113	0.78268
H	37	0.21727	0.00000	0.78161	0.00112	0.78273
* Total *		0.00000	53.97730	121.45313	0.56957	176.00000

NBO Analysis Data (Complex [BeDBSP])

M062X/Def2TZVP

ΔE(multipl.=1) = 0.0 кДж/моль
ΔE(multipl.=3) = 132.2 кДж/моль
Alpha occupied eigenvalues (highest) = -6.3508140 eV
Alpha virtual eigenvalues (lowest) = -1.9860579 eV

<S**2>= 0.0000

Summary of Natural Population Analysis:

			Natural Population			
Atom	No	Natural Charge	Core	Valence	Rydberg	Total
N	1	-0.41092	1.99932	5.38202	0.02959	7.41092
C	2	0.30806	1.99918	3.66775	0.02501	5.69194
C	3	-0.24285	1.99911	4.22566	0.01807	6.24285
N	4	-0.84751	1.99936	5.81317	0.03499	7.84751
C	5	-0.24285	1.99911	4.22566	0.01807	6.24285
C	6	0.30806	1.99918	3.66775	0.02501	5.69194
H	7	0.23219	0.00000	0.76681	0.00100	0.76781
C	8	0.36745	1.99921	3.60961	0.02374	5.63255
C	9	-0.07176	1.99904	4.05275	0.01997	6.07176
N	10	-0.83274	1.99938	5.79891	0.03445	7.83274
C	11	-0.09379	1.99904	4.07477	0.01999	6.09379
C	12	0.41520	1.99920	3.56177	0.02383	5.58480
N	13	-0.46594	1.99933	5.43659	0.03002	7.46594
C	14	0.41520	1.99920	3.56177	0.02383	5.58480
C	15	-0.09380	1.99904	4.07477	0.01999	6.09380
N	16	-0.83274	1.99938	5.79891	0.03445	7.83274
C	17	-0.07176	1.99904	4.05275	0.01997	6.07176
C	18	0.36745	1.99921	3.60961	0.02374	5.63255

H	19	0.23219	0.00000	0.76681	0.00100	0.76781
N	20	-0.41092	1.99932	5.38202	0.02959	7.41092
Be	21	1.67966	1.99828	0.25050	0.07156	2.32034
C	22	-0.17718	1.99913	4.16033	0.01772	6.17718
C	23	-0.17063	1.99913	4.15387	0.01763	6.17063
C	24	-0.19562	1.99926	4.17851	0.01785	6.19562
C	25	-0.20535	1.99925	4.18810	0.01799	6.20535
H	26	0.23095	0.00000	0.76787	0.00118	0.76905
H	27	0.21688	0.00000	0.78218	0.00094	0.78312
H	28	0.21706	0.00000	0.78199	0.00094	0.78294
H	29	0.22994	0.00000	0.76887	0.00120	0.77006
C	30	-0.17063	1.99913	4.15387	0.01763	6.17063
C	31	-0.17718	1.99913	4.16033	0.01772	6.17718
C	32	-0.20535	1.99925	4.18810	0.01799	6.20535
C	33	-0.19562	1.99926	4.17851	0.01785	6.19562
H	34	0.23095	0.00000	0.76787	0.00118	0.76905
H	35	0.22994	0.00000	0.76887	0.00120	0.77006
H	36	0.21706	0.00000	0.78199	0.00094	0.78294
H	37	0.21688	0.00000	0.78218	0.00094	0.78312
* Total *		0.00000	53.97744	121.34383	0.67873	176.00000

NBO Analysis Data (Complex [BeTBSP])

B3PW91/TZVP

ΔE(multipl.=1) = 0.0 кДж/моль
ΔE(multipl.=3) = 126.8 кДж/моль
Alpha occupied eigenvalues (highest) = -5.3307111 eV
Alpha virtual eigenvalues (lowest) = -2.5936572 eV

<S**2>= 0.0000

Summary of Natural Population Analysis:

Atom	No	Natural Charge	Natural Population Core	Valence	Rydberg	Total
N	1	-0.42037	1.99932	5.40539	0.01566	7.42037
C	2	0.35652	1.99916	3.62035	0.02398	5.64348
C	3	-0.08137	1.99904	4.06533	0.01700	6.08137
N	4	-0.80603	1.99940	5.78250	0.02413	7.80603
C	5	-0.08137	1.99904	4.06533	0.01700	6.08137
C	6	0.35653	1.99916	3.62033	0.02398	5.64347
C	7	0.35656	1.99916	3.62030	0.02398	5.64344
C	8	-0.08135	1.99904	4.06531	0.01700	6.08135
N	9	-0.80608	1.99940	5.78255	0.02413	7.80608
C	10	-0.08138	1.99904	4.06534	0.01700	6.08138
C	11	0.35663	1.99916	3.62022	0.02398	5.64337
N	12	-0.42043	1.99932	5.40545	0.01566	7.42043
C	13	0.35664	1.99916	3.62022	0.02398	5.64336
C	14	-0.08138	1.99904	4.06533	0.01700	6.08138
N	15	-0.80610	1.99940	5.78256	0.02413	7.80610
C	16	-0.08135	1.99904	4.06531	0.01700	6.08135
C	17	0.35656	1.99916	3.62030	0.02398	5.64344
N	18	-0.42037	1.99932	5.40540	0.01566	7.42037
Be	19	1.64686	1.99827	0.27268	0.08219	2.35314
C	20	-0.17652	1.99912	4.16353	0.01387	6.17652

C	21	-0.17651	1.99912	4.16352	0.01387	6.17651
C	22	-0.20530	1.99924	4.19183	0.01422	6.20530
C	23	-0.20531	1.99924	4.19184	0.01422	6.20531
H	24	0.22895	0.00000	0.76944	0.00161	0.77105
H	25	0.21642	0.00000	0.78245	0.00113	0.78358
H	26	0.21642	0.00000	0.78245	0.00113	0.78358
H	27	0.22895	0.00000	0.76944	0.00161	0.77105
C	28	-0.17651	1.99912	4.16352	0.01387	6.17651
C	29	-0.17652	1.99912	4.16354	0.01387	6.17652
C	30	-0.20531	1.99924	4.19185	0.01422	6.20531
C	31	-0.20530	1.99924	4.19183	0.01422	6.20530
H	32	0.22895	0.00000	0.76944	0.00161	0.77105
H	33	0.22895	0.00000	0.76943	0.00161	0.77105
H	34	0.21642	0.00000	0.78245	0.00113	0.78358
H	35	0.21642	0.00000	0.78245	0.00113	0.78358
C	36	-0.17652	1.99912	4.16353	0.01387	6.17652
C	37	-0.17652	1.99912	4.16353	0.01387	6.17652
C	38	-0.20532	1.99924	4.19185	0.01422	6.20532
H	39	0.21642	0.00000	0.78245	0.00113	0.78358
C	40	-0.20532	1.99924	4.19185	0.01422	6.20532
H	41	0.21642	0.00000	0.78245	0.00113	0.78358
H	42	0.22895	0.00000	0.76944	0.00161	0.77105
H	43	0.22895	0.00000	0.76944	0.00161	0.77105
* Total *		-0.00000	61.97385	139.39376	0.63239	202.00000

NBO Analysis Data (Complex [BeDBSP])

M062X/Def2TZVP

ΔE(multipl.=1) = 0.0 кДж/моль

ΔE(multipl.=3) = 128.3 кДж/моль

Alpha occupied eigenvalues (highest) = -6.0368106 eV

Alpha virtual eigenvalues (lowest) = -1.8135465 eV

<S**2>= 0.0000

Summary of Natural Population Analysis:

Atom	No	Natural Charge	Natural Population Core	Valence	Rydberg	Total
N	1	-0.43376	1.99932	5.40508	0.02936	7.43376
C	2	0.36947	1.99919	3.60755	0.02379	5.63053
C	3	-0.08463	1.99905	4.06567	0.01992	6.08463
N	4	-0.82682	1.99938	5.79343	0.03401	7.82682
C	5	-0.08458	1.99905	4.06562	0.01992	6.08458
C	6	0.36944	1.99919	3.60758	0.02379	5.63056
C	7	0.36946	1.99919	3.60756	0.02379	5.63054
C	8	-0.08462	1.99905	4.06566	0.01992	6.08462
N	9	-0.82674	1.99938	5.79334	0.03402	7.82674
C	10	-0.08459	1.99905	4.06563	0.01992	6.08459
C	11	0.36946	1.99919	3.60756	0.02379	5.63054
N	12	-0.43376	1.99932	5.40508	0.02936	7.43376
C	13	0.36942	1.99919	3.60760	0.02379	5.63058
C	14	-0.08461	1.99905	4.06565	0.01992	6.08461
N	15	-0.82678	1.99938	5.79339	0.03401	7.82678
C	16	-0.08461	1.99905	4.06565	0.01992	6.08461
C	17	0.36943	1.99919	3.60759	0.02379	5.63057
N	18	-0.43375	1.99932	5.40507	0.02936	7.43375
Be	19	1.68089	1.99828	0.24946	0.07137	2.31911
C	20	-0.17535	1.99913	4.15853	0.01769	6.17535
C	21	-0.17534	1.99913	4.15852	0.01769	6.17534

C	22	-0.20395	1.99926	4.18663	0.01806	6.20395
C	23	-0.20395	1.99926	4.18664	0.01806	6.20395
H	24	0.22872	0.00000	0.77009	0.00119	0.77128
H	25	0.21585	0.00000	0.78321	0.00094	0.78415
H	26	0.21585	0.00000	0.78321	0.00094	0.78415
H	27	0.22872	0.00000	0.77008	0.00119	0.77128
C	28	-0.17535	1.99913	4.15853	0.01769	6.17535
C	29	-0.17535	1.99913	4.15853	0.01769	6.17535
C	30	-0.20394	1.99926	4.18663	0.01806	6.20394
C	31	-0.20394	1.99926	4.18663	0.01806	6.20394
H	32	0.22872	0.00000	0.77008	0.00119	0.77128
H	33	0.22872	0.00000	0.77008	0.00119	0.77128
H	34	0.21585	0.00000	0.78321	0.00094	0.78415
H	35	0.21585	0.00000	0.78320	0.00094	0.78415
C	36	-0.17534	1.99913	4.15852	0.01769	6.17534
C	37	-0.17534	1.99913	4.15852	0.01769	6.17534
C	38	-0.20395	1.99926	4.18664	0.01806	6.20395
H	39	0.21585	0.00000	0.78321	0.00094	0.78415
C	40	-0.20393	1.99926	4.18662	0.01806	6.20393
H	41	0.21585	0.00000	0.78321	0.00094	0.78415
H	42	0.22872	0.00000	0.77009	0.00119	0.77128
H	43	0.22872	0.00000	0.77009	0.00119	0.77128
* Total *		-0.00000	61.97409	139.27487	0.75104	202.00000
